# Tranylcypromine-Based LSD1 Inhibitors as Useful Agents to Reduce Viability of 

**DOI:** 10.1021/acsinfecdis.5c00224

**Published:** 2025-07-02

**Authors:** Emanuele Fabbrizi, Rossella Fioravanti, Clemens Zwergel, Chiara Lambona, Sergio Valente, Giulia Fianco, Angela Iuzzolino, Daniela Trisciuoglio, Jonatan Caroli, Andrea Mattevi, Cécile Häberli, Jennifer Keiser, Dante Rotili, Antonello Mai

**Affiliations:** † Department of Drug Chemistry and Technologies, 9311 Sapienza University of Rome, Piazzale Aldo Moro 5, 00185 Rome, Italy; ‡ School of Pharmacy, 128165 Mekelle University, Mekelle 0231, Ethiopia; § School of Pharmacy, 6106 University of East Anglia, Norwich NR4 7TJ, U.K.; ∥ Institute of Molecular Biology and Pathology, 9327 National Research Council (CNR), Rome 00185, Italy; ⊥ Department of Biology and Biotechnology, University of Pavia, Via Ferrata 9, Pavia 27100, Italy; # Swiss Tropical and Public Health Institute, University of Basel, 4002 Allschwil, Switzerland, 4001 Basel, Switzerland; ¶ Department of Science, Roma Tre University of Rome, Viale Guglielmo Marconi 446, 00146 Rome, Italy; ∇ Biostructures and Biosystems National Institute (INBB), Via dei Carpegna 19, 00165 Rome, Italy

**Keywords:** lysine demethylases, LSD1, *S. mansoni*, reduction of viability, cytotoxicity

## Abstract

infections remain a major public health issue mainly in tropical and subtropical regions. While Praziquantel is the primary treatment for schistosomiasis, its limitations include resistance development and poor efficacy against juvenile worms. Given the biological similarities between tumor and parasite-infected cells, LSD1 inhibitors—primarily explored as anticancer agents—have been investigated for their antiparasitic potential.

## Graphical abstract

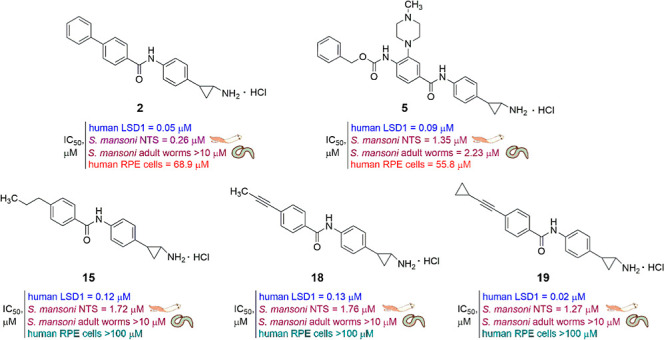

Recently, we identified MC3935 as an LSD1 inhibitor active against . Encouraged by its potential, we selected 13 LSD1 inhibitors from our library (**1**–**13**) and synthesized nine MC3935 analogs (**14**–**22**) for the evaluation against newly transformed schistosomula (NTS) and adult worms. Compounds **2**, **5**, **15**, **18**, and **19** exhibited potent activity against NTS, with the last four also showing viability reduction against adult worms at 20 μM. Cytotoxicity tests confirmed selective efficacy for **15**, **18**, and **19**. These findings identify such three LSD1 inhibitors as new, valuable starting points for the treatment of schistosomiasis.

 is a parasitic trematode responsible for intestinal schistosomiasis, a major public health concern in tropical and subtropical regions. This species is prevalent in sub-Saharan Africa, Middle East, Caribbean, and South America.^1^

The life cycle of involves freshwater snails of the genus as intermediate hosts. Humans become infected through contact with contaminated water, allowing the parasite’s larvae to penetrate the skin. Once inside the host, the larvae mature into adult worms residing in the mesenteric veins, where they produce eggs. Some of these eggs are excreted in feces, continuing the transmission cycle, while others become trapped in host tissues, triggering inflammatory responses and causing organ damage.^2^

Clinical manifestations of infection span from acute symptoms such as fever and abdominal pain to chronic conditions, including liver fibrosis and portal hypertension. The World Health Organization estimates that at least 250 million people were affected by schistosomiasis in 2021.^3^

Efforts to control infections focus on preventive chemotherapy, snail control, improved sanitation, and public health education to reduce transmission and associated morbidity. For decades, the first-line treatment for schistosomiasis has been Praziquantel, a pyrazino-isoquinoline derivative effective against the adult form of the parasite.^4–6^ However, prolonged administration of Praziquantel has led to concerns regarding reduced efficacy due to the emergence of resistant strains.^7,8^ Furthermore, Praziquantel shows limited effectiveness against juvenile stages, highlighting the need for novel therapeutic approaches.

Following the sequencing of the genome, the search for new therapeutic agents has gained renewed momentum, driven by the identification of alternative drug targets. Small-molecule modulators of epigenetic pathways regulate gene transcription by altering chromatin structure, switching between gene activation and silencing.^9^ The primary clinical application of epigenetic drugs (epi-drugs) has been in cancer chemotherapy,^10^ and several have already been approved for oncological use.^11^ Since there are some similarities between parasites and cancer cells^12^—including increased metabolic activity,^13^ immune evasion mechanisms,^14^ and shared therapeutic targets,^15^ together with some differences as well,^13,14^ —our group has tested histone deacetylase (HDAC) and sirtuin inhibitors from our library against .^16–18^ Some of these inhibitors specifically targeted the parasite epigenetic enzyme *Sm*HDAC8, while others exhibited *Sm*SIRT2 inhibitory activity.

In addition to (de)­acetylation, the histone lysine (de)­methylation plays a crucial role in epigenetic regulation. Lysine specific demethylase 1 (LSD1, KDM1A) removes methyl groups from histone H3 lysine 4 or 9 (H3K4 or H3K9), leading to transcriptional activation or repression, respectively. Due to this dual function, LSD1 regulates key biological processes, including cell differentiation, development, and tumorigenesis, making it a critical factor in both physiological and pathological conditions.^19,20^ LSD1 is overexpressed in various cancers, including leukemia, lung cancer, and breast cancer, and several small-molecule LSD1 inhibitors (LSD1i) are currently in clinical trials for cancer treatment.^21^

Since 2010, we have been working to identify small molecules able to inhibit LSD1, including both covalent inhibitors—based on the structure of tranylcypromine (TCP), a monoamine oxidase (MAO) inhibitor that also inhibits LSD1—and reversible inhibitors. During these years, we have developed various compounds with potent anticancer activity.^22–33^ During a study on TCP-based functionalized probes for activity-based protein profiling of LSD1,^34^ we discovered that unsaturated side-chains on the TCP scaffold are fully compatible with LSD1 inhibition. One such compound, MC3935 (Figure 1), was recently described by us as a potent human LSD1i with profound effects on , further validating LSD1 as a viable epigenetic target for treating this parasitic infection.^35,36^ Using in silico techniques, we showed that MC3935 binds to the LSD1 (*Sm*LSD1) catalytic pocket, and knockdown of *Sm*LSD1 by RNAi phenocopied the MC3935 effects in adult worms.^35^ Treatment of juvenile and adult worms with MC3935 led to severe tegument damage, impaired egg production, and parasite death within 96 h. Moreover, transcriptomic analysis of MC3935-treated parasites revealed alterations in the expression of hundreds of genes involved in key biological processes.^35^

**Figure 1:**
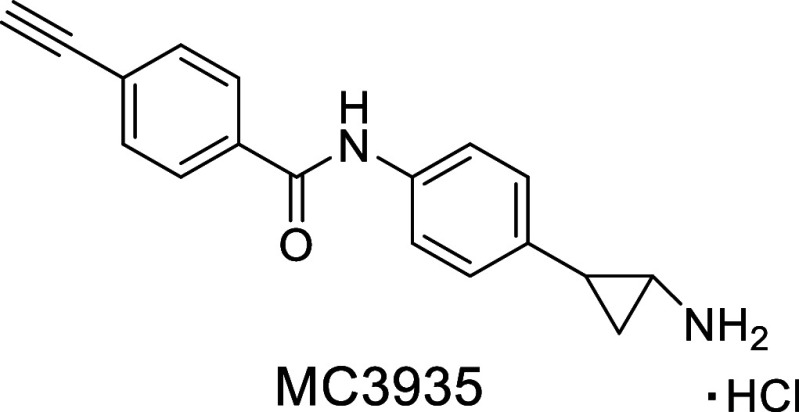
Chemical structure of MC3935.

Based on these findings, this study aimed first to test a selection of 13 TCP-based LSD1i (compounds **1**–**13**, Figure 2) from our in-house library against [newly transformed schistosomula (NTS) and adult worms]. Then, we synthesized and evaluated a small series of new MC3935 analogs containing (un)­saturated side chains at the TCP phenyl ring (compounds **14**–**22**, Figure 3) using the same models.

**Figure 2:**
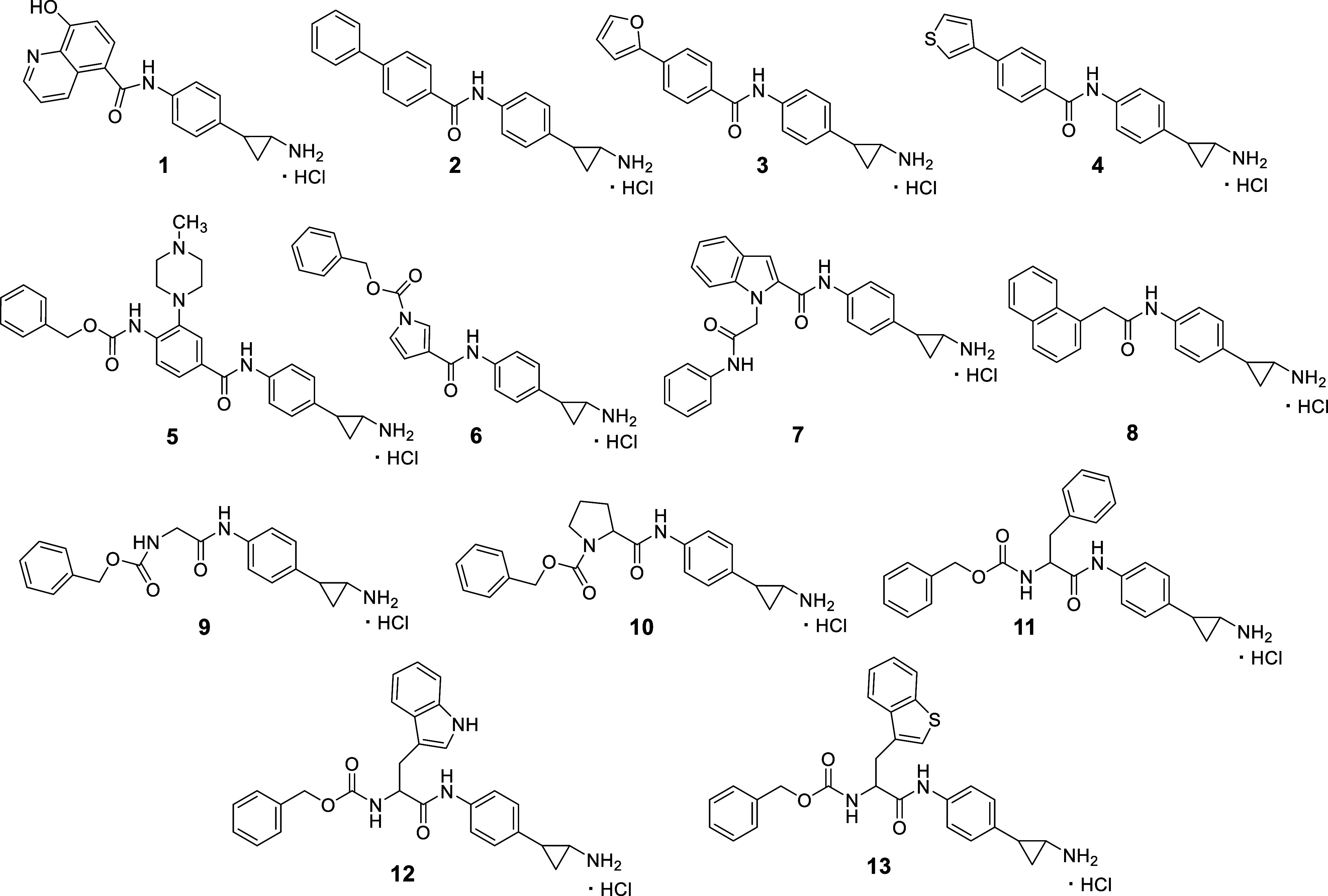
TCP-based LSD1 inhibitors selected from our in-house library.

**Figure 3:**
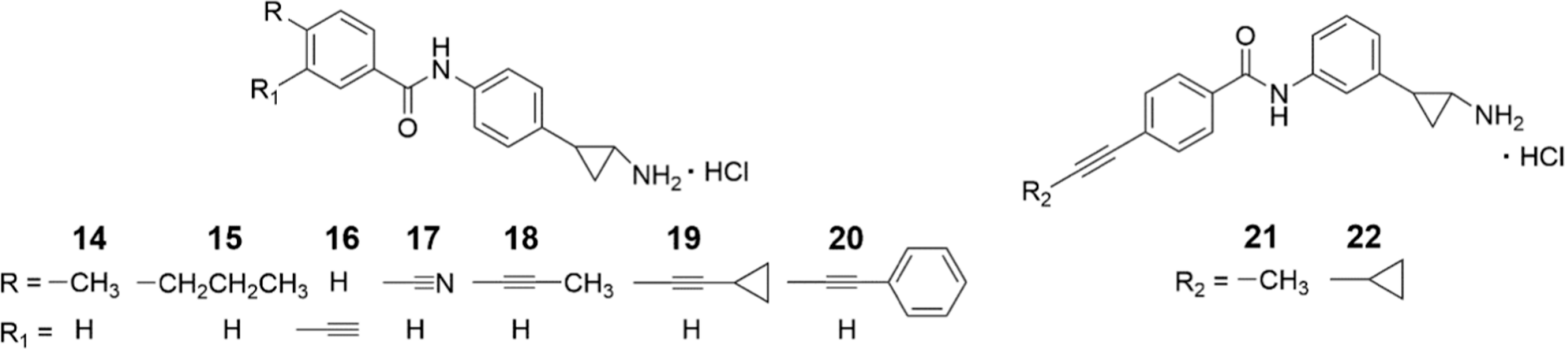
Newly prepared MC3935 analogs **14**–**22** bearing or not unsaturated chains at the TCP phenyl ring.

## Results

### Chemistry

The synthesis of the new compounds **14**–**22** was carried out according to Scheme 1. The intermediate *tert*-butyl [2-(4-aminophenyl)­cyclopropyl]­carbamate (**23**)^22^ was treated with the appropriate benzoyl chlorides (**24**–**29**), either commercially available (**24**–**26**) or synthesized in-house (**27**–**29**, see Scheme 2), in the presence of triethylamine (TEA) in dry dichloromethane, yielding the benzoylamino-carbamates **30**–**35** (Scheme 1A). The coupling reaction of **23** with the commercial 3-ethynylbenzoic acid (**36**) using 1-ethyl-3-(3-(dimethylamino)­propyl)­carbodiimide hydrochloride (EDCI) and hydroxybenzotriazole (HOBt) in dry DMF afforded the 3-ethynylbenzoylamino-carbamate (**37**) (Scheme 1A). Similarly, treatment of the *tert*-butyl [2-(3-aminophenyl)­cyclopropyl]­carbamate (**38**)^24^ with either the 3-(prop-1-yn-1-yl)­benzoyl chloride (**27**) or 4-(cyclopropylethynyl)­benzoyl chloride (**28**) in the presence of TEA produced the corresponding carbamates **39** and **40** (Scheme 1B). All key carbamate intermediates (**30**–**35**, **37**, **39**, **40**) underwent Boc deprotection via 4 N hydrochloric acid in dioxane using tetrahydrofuran as a solvent, yielding the final compounds **14**–**22** as hydrochlorides.

**Scheme 1:**
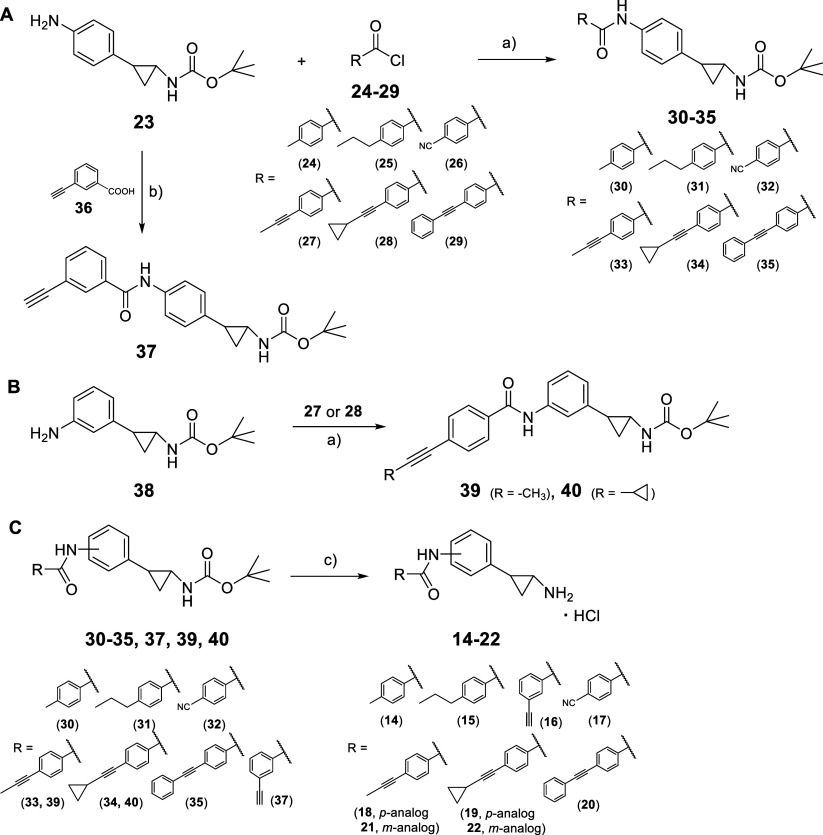
Synthetic Routes for the Novel LSD1 Inhibitors **14–22** ^a^

a Conditions: (a) TEA, dry DCM, 2–4 h, 0 °C to rt; (b) EDCI, HOBT, TEA, DMF, N_2_, 7 h, rt; (c) 4 N HCl in dioxane, dry THF, overnight, rt.

**Scheme 2:**
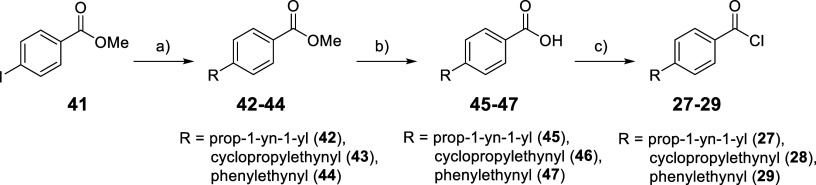
Synthesis of the Benzoyl Chlorides **27–29** ^a^

a Conditions: (a) appropriate alkyne, 5% PdCl_2_(PPh_3_)_2_, 5% CuI, TEA, dry THF/DMF, overnight, rt to 70 °C; (b) 2 N LiOH, MeOH:H_2_O (3:1), overnight, rt; (c) SOCl_2_, 3 h, reflux.

The benzoyl chloride intermediates (**27**–**29**) were synthesized starting from the commercially available methyl 4-iodobenzoate (**41**), which was subjected to Sonogashira coupling with the appropriate alkynes using bis­(triphenylphosphine)­palladium­(II) dichloride [PdCl_2_(PPh_3_)_2_] and copper­(I) iodide (CuI) in dry DMF/THF, yielding the corresponding methyl 4-alkynyl-benzoates (**42**–**44**). These methyl esters were hydrolyzed using aqueous 2 N lithium hydroxide in methanol under reflux to obtain the corresponding carboxylic acids (**45**–**47**). Finally, refluxing **45**–**47** with an excess of thionyl chloride generated the desired benzoyl chlorides (**27**–**29**) (Scheme 2).

### In Vitro Human LSD1-CoREST Inhibition

Compounds **14**–**22** were tested against the human LSD1-CoREST complex to determine their inhibitory activity. Spectrophotometric analysis revealed bleaching of the flavin cofactor by **14**–**20**, whereas no bleaching was observed for **21** and **22**, indicating a lack of inhibition. ThermoFAD assays were then performed to assess the impact of **14**–**22** on the thermal stability (Δ*T*_m_ values, Table 1) of the LSD1-CoREST complex.^37^ The most significant shifts in *T*m values corresponded to the strongest binders. Consistent with the spectrophotometric data, compounds **14**–**20** stabilized the protein, whereas **21** and **22** showed no thermal shift compared to the control (Figure 4). To confirm these findings, horseradish peroxidase (HRP)-coupled LSD1 inhibition assays were conducted on **14**–**21**, comparing them with TCP and MC3935 as positive controls (IC_50_ values, Table 1) (Figure 5). Compound **22** was not tested, considering its previous negative results.

**Table 1:** Evaluation of hLSD1 Binding Capability and hLSD1 Direct Inhibition by **14–22**

compd	Δ*T*_m_, °C	IC_50_, μM^a^
**14**	+8	0.15 ± 0.02
**15**	+9.5	0.12 ± 0.02
**16**	+10	0.07 ± 0.02
**17**	+8.5	0.15 ± 0.02
**18**	+8	0.13 ± 0.02
**19**	+8	0.02 ± 0.001
**20**	+8	4.02 ± 0.41
**21**	0	>100
**22**	0	ND^b^
MC3935		0.52^c^
TCP		11.2 ± 1.8

a The IC_50_ values reported are based on two separate curves, wherein each data point is the average of two determinations. The resulting IC_50_ values from these curves were then averaged and reported in the table. The error is within ±10%.

b ND, not determined.

c Ref 35.

**Figure 4:**
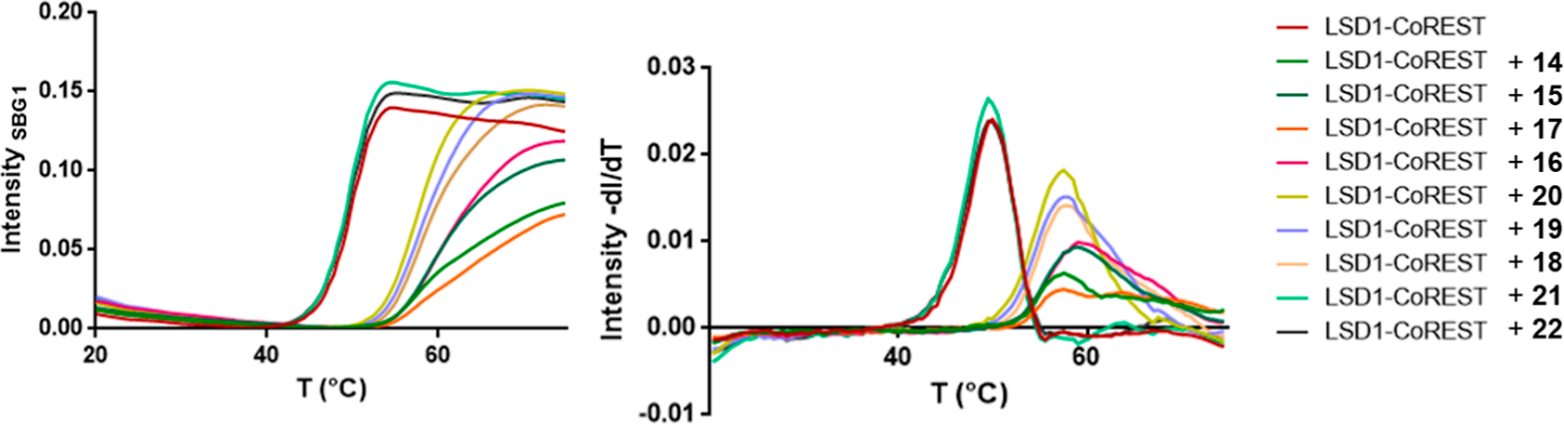
ThermoFAD assay performed on **14**–**22**. Compounds **14**–**20** showed similar binding activity, whereas **21** and **22** were clearly inactive.

**Figure 5:**
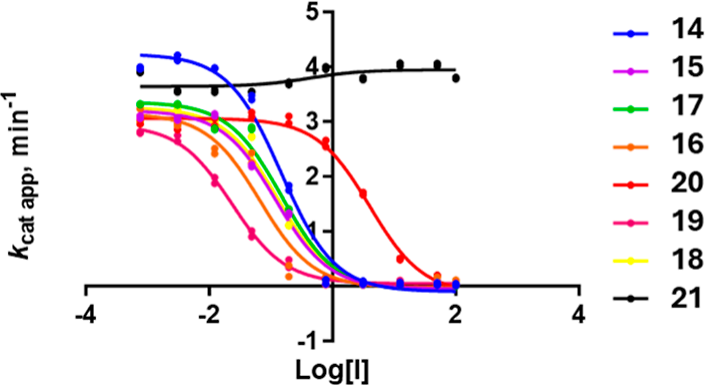
HRP-coupled hLSD1 inhibition assays performed on **14**–**21**.

The *N*-(4-(2-aminocyclopropyl)­phenyl)-3- or −4-substituted benzamides **14**–**19** inhibited LSD1-CoREST with IC_50_ values between 0.02 and 0.15 μM. Compound **20** exhibited single-digit μM inhibition, whereas **21** was inactive, further validating the spectrophotometric and ThermoFAD results, and confirming the >100-fold decrease of potency observed before by us, comparing the benzyl (1-((4-(2-aminocyclopropyl)­phenyl)­amino)-1-oxo-3-phenylpropan-2-yl)­carbamate (**11**, IC_50_ = 0.15 μM) with the corresponding 3-(2-aminocyclopropyl)­phenyl *meta* analog (IC_50_ = 18.1 μM).^27^

### Evaluation of TCP-Based LSD1 Inhibitors **1–20** against NTS and Adult Worms

To assess the effects of covalent LSD1i against , we tested against the parasite 13 selected TCP-based compounds from our in-house library (**1**–**13**, Figure 2), along with the newly synthesized compounds **14**–**20** (Figure 3). The compounds **1**–**13** feature a TCP moiety substituted at the C4-position of the phenyl ring with different functional groups: an amino group linked to an aroyl portion (**1**–**4**,^27–29^ **6**,^31^ **7** ^31^), a benzoyl-benzyl carbamate function (**5**),^27^ a 1-naphthylacetyl group (**8**),^27^ or a *Z*-amino acid (**9**–**13**).^22,27^ When incubated with the human recombinant LSD1-CoREST enzymatic complex, **1**–**13** showed submicromolar to nanomolar potency toward LSD1 inhibition, except for compound **3**,^28^ which had an IC_50_ of 2 μM (Table S1 in Supporting Information).

Compounds **1**–**20** were screened against NTS at 20 and 10 μM for 72 h. Those showing ≥50% activity at 10 μM were further tested at 1 and 0.1 μM for IC_50_ determination (Table 2). MC3935 was included for comparison.

**Table 2:** Reduction of NTS Viability by **1**–**20**

compd	Percentage of reduction of NTS viability, 72 h^a^				IC_50_ (μM)^b^
	20 μM	10 μM	1 μM	0.1 μM	
**1**	45.8 ± 4.2	37.9 ± 0.0	ND^c^	ND	>20
**2**	100 ± 0.0	98 ± 1.0	56.9 ± 0.9	43.7 ± 2.1	0.26
**3**	37.50 ± 0.0	36.2 ± 0.9	ND	ND	>20
**4**	79.17 ± 0.0	43.1 ± 0.9	ND	ND	>10
**5**	100 ± 0.0	100 ± 0.0	18.0 ± 1.0	ND	1.35
**6**	100 ± 0.0	40.0 ± 2.0	ND	ND	>10
**7**	66.7 ± 4.2	37.9 ± 0.0	ND	ND	>10
**8**	50.0 ± 0.0	46.5 ± 2.6	ND	ND	>10
**9**	41.7 ± 4.2	37.93 ± 1.7	ND	ND	>20
**10**	37.5 ± 4.2	32.8 ± 0.9	ND	ND	>20
**11**	100 ± 0.0	51.7 ± 0.0	25.0 ± 0.0	ND	2.13
**12**	50.0 ± 0.0	41.4 ± 1.7	ND	ND	>10
**13**	50.0 ± 4.2	36.2 ± 0.9	ND	ND	>10
**14**	41.7 ± 4.2	22.9 ± 2.1	ND	ND	>20
**15**	100 ± 0.0	87.5 ± 4.2	30.2 ± 1.9	ND	1.72
**16**	77.8 ± 2.1	31.2 ± 6.3	ND	ND	>10
**17**	31.2 ± 6.3	25.0 ± 0.0	ND	ND	>20
**18**	100 ± 0.0	95.8 ± 4.2	22.6 ± 1.9	ND	1.76
**19**	100 ± 0.0	100 ± 0.0	22.6 ± 1.9	ND	1.27
**20**	77.8 ± 2.1	29.2 ± 0.0	ND	ND	>10
MC3935	100 ± 0.0	45.5 ± 4.3	33.3 ± 0.0	ND	1.95

a The number of replicates is at least *n* = 2.

b IC_50_, compound concentration that inhibits 50% of the viability of the parasites.

c ND, not determined.

The most potent compounds against NTS were **2**, **5**, **11**, **15**, **18**, and **19**, with IC_50_ values ranging from 0.26 to 2.13 μM. Differently from what was observed in the case of some *Sm*HDAC8 inhibitors,^17,38^ there is approximately a linear correlation between anti-LSD1 potency and *in vitro* effectiveness in the reduction of viability on schistosomes, with the correlation index = 0.753 (Figure S1 in Supporting Information). Notably, the 4-benzoylamino TCP derivatives **2**, **5**, **15**, **18**, and **19**, regardless of the substitution at the benzoyl ring, exhibited the highest death induction in NTS, with **2** being the most effective, while the *Z*-Phe-based analog **11** was the least potent. Among the newly described compounds, **15**, **18**, and **19** displayed similar potencies between them and MC3935, with **19** (IC_50_ = 1.27 μM) being the most active. The shift of the ethynyl group of MC3935 from para to meta position at the benzamide portion (compound **16**), as well as the replacement of the same ethynyl group by the cyano one (compound **17**) or the introduction of the bulky phenylethynyl group (compound **20**) clearly reduced the antischistosomal effect (Table 2).

Afterward, a subset of active compounds **2**, **4**–**7**, **11**, **15**, **16**, **18**–**20** were tested against adult worms at 20 μM for 72 h (Table 3). Among them, **2**, **4**, **5**, **15**, **18**, and **19** reduced worm viability >50%. Notably, the last four of them displayed 78–89% of death induction at 20 μM, with compound **5** demonstrating the highest potency (IC_50_ = 2.23 μM). While **2** was the most effective against NTS, its potency dropped a lot in adult worms.

**Table 3:** Effect of Selected LSD1 Inhibitors on Adult Worm Viability Reduction

compd	adult worms’ percentage of activity, 72 h^a^			IC_50_ (μM)^b^
	20 μM	10 μM	1 μM	
**2**	51.9 ± 0.0	39.2 ± 2.0	ND^c^	>10
**4**	55.6 ± 0.0	43.3 ± 3.3	ND	>10
**5**	88.9 ± 0.0	56.9 ± 0.0	40.7 ± 7.4	2.23
**6**	31.3 ± 1.9	ND	ND	>20
**7**	40.7 ± 3.7	ND	ND	>20
**11**	44.4 ± 0.0	ND	ND	>20
**15**	78.1 ± 7.3	39.3 ± 4	ND	>10
**16**	39.6 ± 0.0	ND	ND	>20
**18**	87.5 ± 0.0	41.4 ± 0.2	ND	>10
**19**	81.25 ± 6.3	34.7 ± 5.3	ND	>10
**20**	43.9 ± 1.9	ND	ND	>20
MC3935	88.9 ± 0.0	41.6 ± 1.7	ND	>10

a The number of replicates is at least *n* = 2.

b IC_50_, compound concentration that inhibits 50% of the viability of the parasites.

c ND, not determined.

Selected compounds **2**, **15**, **18**, and **19** were tested on adult worms to evaluate their effects on pairing and egg laying. While no impact on worm pairing was observed for any of the tested compounds, a reduction in egg laying was detected at 4, 8, 24, 48, and 72 h following treatment with 20 μM, starting as early as 4 h after exposure (Table 4).

**Table 4:** Effects of Selected LSD1 Inhibitors (20 μM) on Egg Laying

compd	eggs’ number				
	4 h	8 h	24 h	48 h	72 h
ctr	0	0	23	89	105
**2**	0	0	0	0	6
**15**	0	0	0	0	8
**18**	0	0	0	0	2
**19**	0	0	0	0	5

### Speed-of-Action Studies in NTS and Juvenile Forms

To better characterize a selection of our LSD1i *in vitro*, we assessed the onset of their action, defined as the time required for an observable antischistosomal effect. To evaluate the rapidity with which a compound impacts parasite viability, we conducted speed-of-action assays by determining the IC_50_ values at different time points (4, 24, 48, and 72 h post drug exposure), in both NTS and juvenile forms (Tables 5 and 6). For detailed percentages of activity at each time point, refer to the Supporting Information (Tables S2 and S3 in Supporting Information). Against NTS, compounds began to exhibit activity after 24 h, which progressively increased and reached its peak at 72 h. In this assay, compounds **15**, **18**, and **19** - although chemically related to MC3935 - showed an earlier onset and greater potency compared to the prototype. When tested against juvenile worms, all compounds demonstrated a delayed onset of action, with observable activity only at the 72 h time point. In this case as well, **15**, **18**, and **19** were 4- to 7-fold more effective than MC3935.

**Table 5:** Speed-of-Action Data for Antischistosomal Activity in NTS

compd	IC_50_, μM			
	4 h	24 h	48 h	72 h
**2**	>20	5.0	4.0	2.3
**15**	>20	10.0	9.9	9.3
**18**	>20	6.9	4.4	2.4
**19**	>20	10.4	8.3	7.5
MC3935	>20	>20	17.6	11.5

**Table 6:** Speed-of-Action Data for Antischistosomal Activity in Juvenile Forms

compd	IC_50_, μM			
	4 h	24 h	48 h	72 h
**2**	>20	16.2	>20	5.9
**15**	>20	>20	>20	4.3
**18**	>20	>20	>20	3.8
**19**	>20	>20	>20	2.7
MC3935	>20	>20	>20	17.7

### Cytotoxic Activity of Selected LSD1 Inhibitors in Human Cells

LSD1i often exhibit potent anticancer effects and may, therefore, pose a risk of toxicity to human cells, including noncancerous ones. To assess their cytotoxicity, we tested the compounds identified as the most effective against the parasite (**2**, **5**, **15**, **18**, and **19**) in two human cell lines, the retinal pigment epithelial (RPE) and the MRC-5 fibroblast cells at concentrations of 5, 10, 25, and 50 μM. The MTT method was used to evaluate cell viability after 72 h of treatment. Based on data reported in Table 7, compounds **2** and **5** displayed high to moderate toxicity, while compounds **15**, **18**, and **19**—similarly to MC3935—did not significantly affect viability in both cell lines, even at the highest tested dose (50 μM).

**Table 7:** Cytotoxic Effect of Selected LSD1 Inhibitors in Human RPE Cells

	RPE cells					MRC-5 cells				
compd	percentage of viability, 72 h				IC_50_, μM	percentage of viability, 72 h				IC_50_, μM
	5 μM	10 μM	25 μM	50 μM		5 μM	10 μM	25 μM	50 μM	
**2**	95 ± 9	82 ± 11	87 ± 39	49 ± 9	68.9	29 ± 2	26 ± 3	13 ± 3	10 ± 2	5.9
**5**	63 ± 33	72 ± 18	82 ± 11	57 ± 10	55.8	75 ± 7	59 ± 5	58 ± 2	39 ± 4	24.3
**15**	89 ± 3	104 ± 12	99 ± 6	106 ± 17	>100	81 ± 16	91 ± 20	89 ± 17	92 ± 20	>100
**18**	82 ± 21	86 ± 36	104 ± 20	95 ± 2	>100	111 ± 10	75 ± 15	98 ± 14	75 ± 12	>100
**19**	92 ± 26	89 ± 35	98 ± 21	103 ± 20	>100	98 ± 20	88 ± 18	85 ± 8	85 ± 35	>100
MC3935	84 ± 18	87 ± 24	82 ± 11	100 ± 33	>100	94 ± 10	97 ± 9	93 ± 17	94 ± 10	>100

## Discussion and Conclusions

LSD1i are epigenetic compounds primarily explored as anticancer agents, with some of them currently in clinical trials.^21^ Given the biological similarities between tumor and parasite-infected cells, such as metabolic adaptations,^39^ immune evasion strategies,^40^ and mechanisms of cellular invasion,^41^ it is reasonable to investigate epi-drugs for their potential antiparasitic effects, prioritizing those with the broadest therapeutic window.

Over the past 15 years, we have been developing numerous LSD1i, both irreversible and reversible, many of which exhibit anticancer activity.^22–33^ During our research on chemical probes for activity-based protein profiling of LSD1 activity,^34^ we identified a TCP-based intermediate compound, MC3935 (Figure 1), which demonstrated anti-LSD1 activity and potential efficacy against infection.^35^ Encouraged by these findings, we selected 13 structurally diverse LSD1i (compounds **1**–**13**, Figure 2) from our in-house library and synthesized nine new MC3935 analogs (compounds **14**–**22**, Figure 3) for evaluation against NTS and adult worms. Most of the compounds **14**–**22** featured unsaturated chains at the TCP phenyl ring, similar to MC3935. Among them, seven (**14**–**20**) were substituted at the para position of the TCP phenyl ring, while two (**21**, **22**) were at the meta position.

When tested against the human LSD1-CoREST complex, compounds **14**–**20** showed excellent to good LSD1 inhibition. In contrast, the meta-analogs **21** and **22** were inactive, confirming our previous findings regarding this regioisomerism.^27^ Compounds **1**–**20** were then tested against NTS and adult worms to assess their impact on parasite viability. While against NTS some compounds (**2**, **5**, **11**, **15**, **18**, and **19**) exhibited high potency, against adult worms most of them displayed reduced efficacy, similarly to what observed with other epi-drugs such as HDAC and sirtuin inhibitors.^16–18^ Only compound **5** retained its potency in adult worms with an IC_50_ of 2.23 μM. Our results confirm that NTS activity does not always correlate with killing of adult worms but, given the limitation in supply of adult worms, this stage is useful as screening tool in particular for large libraries of compounds.^42^

The speed-of-action assay in is crucial for early stage evaluation in drug development, helping to identify compounds with rapid schistosomicidal activity. By determining how quickly a compound exerts its effects on the parasite, researchers can prioritize fast acting compounds, which may translate to enhanced efficacy in *in vivo* studies as a long half-life is not needed.^42,43^

Some of the most potent compounds against NTS (**2**, **15**, **18**, and **19**) were tested in speed-of-action assays on both NTS and juvenile forms. Compounds showed activity after 24 h (NTS) and 72 h (juvenile worms). The differences in activity might be explained by the physiological and biochemical differences between the two worm stages. Though the compounds cannot be considered as fast acting, in a next step *in vivo* study should be launched to evaluate the most promising candidates in the rodent model.

To assess cytotoxicity and parasite selectivity, compounds **2**, **5**, **15**, **18**, and **19** were tested in human RPE and MRC-5 cells. Among the tested compounds, **2** and **5** showed high to moderate viability reduction, where as **15**, **18**, and **19**, as well as MC3935, exhibited low cytotoxicity even at the highest tested dose (50 μM).

In conclusion, despite some limitations of this study, including the exclusive use of in vitro assays, potential challenges in drug delivery for LSD1 inhibitors, and possible impacts on the host’s metabolic system, our findings confirm LSD1 as a promising target for infection, and identify compounds **15**, **18**, and **19** as potential chemotherapeutic starting points for schistosomiasis treatment.

## Methods

### Chemistry

Melting points were determined using a Buchi 530 melting point apparatus. ^1^H-NMR spectra were recorded at 400 MHz on a Bruker AC 400 spectrometer, with chemical shifts reported in δ (parts per million, ppm) units relative to the internal reference tetramethylsilane (Me_4_Si). Mass spectra were recorded on an API-TOF Mariner by Perspective Biosystem (Stratford, TX), with samples injected by a Harvard pump at a flow rate of 5–10 μL/min, infused in the electrospray system. All compounds were routinely analyzed using thin-layer chromatography (TLC) and ^1^H-NMR. TLC was performed on aluminum-backed silica gel plates (Merck DC, Alufolien Kieselgel 60 F_254_), with spot visualization under UV light or using an alkaline KMnO_4_ solution. All solvents were reagent-grade and, when necessary, were purified and dried by standard methods. Reaction and extraction solutions were concentrated using a rotary evaporator under reduced pressure (∼20 Torr). Organic solutions were dried over anhydrous sodium sulfate. Elemental analysis confirmed that the compounds described had a purity of >95%, with analytical results deviating by no more than 0.40% from theoretical values (Table S4 in Supporting Information, determined on the free bases). All chemicals were purchased from Sigma-Aldrich s.r.l. (Milan, Italy) or from TCI Europe N.V. (Zwijndrecht, Belgium) and were of the highest purity. Samples prepared for physical and biological studies were routinely dried under high vacuum over P_2_O_5_ for 20 h at temperatures ranging from 25 to 40 °C, depending on the sample’s melting point.

#### General Procedure for the Preparation of the *N*-(3- or 4-(2-aminocyclopropyl)­phenyl)-3- or 4-(substituted)­benzamides **14–22**

##### Example: *N*-(4-(2-Aminocyclopropyl)­phenyl)-4-cyanobenzamide Hydrochloride (**17**)

*tert*-Butyl (2-(4-(4-cyanobenzamido)­phenyl)­cyclopropyl)­carbamate **32** (0.253 mmol, 95.5 mg, 1.0 equiv) was dissolved in dry THF (6 mL) and the solution was stirred at 0 °C. Then, 4 N HCl in 1,4-dioxane (16.45 mmol, 4.1 mL, 65 equiv) was added dropwise, and the mixture was allowed to warm at rt. After 48 h, when the conversion was complete, the suspension was filtered and washed with dry THF and then with dry Et_2_O to afford **17** as a hydrochloride salt (white solid; yield, 65%). mp >250 °C. Recrystallization solvent: methanol. ^1^H-NMR (DMSO-*d*_6_, 400 MHz): δ_H_/ppm 1.17–1.22 (m, 1H, −CH*H*–cyclopropane), 1.36–1.38 (m, 1H, −C*H*H–cyclopropane), 2.32–2.34 (m, 1H, Ar–C*H*–cyclopropane), 2.77–2.82 (m, 1H, -C*H*-NH_3_^+^), 7.16 (d, 2H, benzene ring), 7.71 (d, 2H, benzene ring), 8.03 (d, 2H, benzene ring), 8.11 (d, 2H, benzene ring), 8.46 (bs, 3H, –N*H*_3_^+^), 10.51 (bs, 1H, −CO–N*H*-Ar). MS (ESI), *m*/*z*: 278 [M + H]^+^.

##### *N*-(4-(2-Aminocyclopropyl)­phenyl)-4-methylbenzamide Hydrochloride (**14**)

White solid; yield, 86%. mp >250 °C. Recrystallization solvent: methanol. ^1^H-NMR (DMSO-*d*_6_, 400 MHz): δ_H_/ppm 1.16–1.22 (m, 1H, −CH*H*-cyclopropane), 1.34–1.39 (m, 1H, −C*H*H-cyclopropane), 2.28–2.33 (m, 1H, Ar–C*H*-cyclopropane), 2.39 (s, 3H, −C*H*_3_), 2.77–2.80 (m, 1H, -C*H*-NH_3_^+^ cyclopropane), 7.14 (d, 2H, benzene ring), 7.33 (d, 2H, benzene ring), 7.71 (d, 2H, benzene ring), 7.87 (d, 2H, benzene ring), 8.42 (bs, 3H, -N*H*_3_^+^), 10.15 (bs, 1H, −CO–N*H*-Ar). MS (ESI), *m*/*z*: 267 [M + H]^+^.

##### *N*-(4-(2-Aminocyclopropyl)­phenyl)-4-propylbenzamide Hydrochloride (**15**)

White solid; yield, 79%. mp >250 °C. Recrystallization solvent: methanol. ^1^H-NMR (DMSO-*d*_6_, 400 MHz): δ_H_/ppm 0.90 (t, 3H, –CH_2_CH_2_C*H*_3_), 1.16–1.21 (m, 1H, −CH*H*-cyclopropane), 1.33–1.38 (m, 1H, −C*H*H-cyclopropane), 1.57–1.67 (m, 2H, −CH_2_C*H*_2_CH_3_), 2.27–2.32 (m, 1H, Ar–C*H*-cyclopropane), 2.63 (t, 2H, −C*H*_2_CH_2_CH_3_), 2.76–2.80 (m, 1H, -C*H*-NH_3_^+^), 7.13 (d, 2H, benzene ring), 7.34 (d, 2H, benzene ring), 7.70 (d, 2H, benzene ring), 7.87 (d, 2H, benzene ring), 8.38 (bs, 3H, −N*H*_3_^+^), 10.15 (bs, 1H, −CO–N*H*-Ar). MS (ESI), *m*/*z*: 295 [M + H]^+^.

##### *N*-(4-(2-Aminocyclopropyl)­phenyl)-3-ethynylbenzamide Hydrochloride (**16**)

White solid; yield, 70%. mp >250 °C. Recrystallization solvent: methanol. ^1^H-NMR (DMSO-*d*_6_, 400 MHz): δ_H_/ppm 1.16–1.21 (m, 1H, −CH*H*-cyclopropane), 1.34–1.40 (m, 1H, −C*H*H-cyclopropane), 2.28–2.32 (m, 1H, Ar–C*H*-cyclopropane), 2.76–2.79 (m, 1H, -C*H*–NH_3_^+^), 4.33 (s, 1H, −C*H* acetylene), 7.15 (d, 2H, benzene ring), 7.55 (t, 1H, benzene ring), 7.70 (t, 3H, benzene ring), 7.97 (d, 1H, benzene ring), 8.05 (s, 1H, benzene ring), 8.42 (bs, 3H, –N*H*_3_^+^), 10.33 (bs, 1H, −CO–N*H*-Ar). MS (ESI), *m*/*z*: 277 [M + H]^+^.

##### *N*-(4-(2-Aminocyclopropyl)­phenyl)-4-(prop-1-yn-1-yl)­benzamide Hydrochloride (**18**)

White solid; yield, 78%. mp >250 °C. Recrystallization solvent: methanol. ^1^H-NMR (DMSO-*d*_6_, 400 MHz): δ_H_/ppm 1.16–1.21 (m, 1H, −CH*H*-cyclopropane), 1.34–1.38 (m, 1H, −C*HH*-cyclopropane), 2.09 (s, 3H, −C*H*_3_), 2.31–2.33 (m, 1H, Ar–C*H*-cyclopropane), 2.77–2.79 (m, 1H, -C*H*-NH_3_^+^), 7.14 (d, 2H, benzene ring), 7.53 (d, 2H, benzene ring), 7.70 (d, 2H, benzene ring), 7.92 (d, 2H, benzene ring), 8.40 (bs, 3H, –N*H*_3_^+^), 10.27 (bs, 1H, −CO–N*H*-Ar). MS (ESI), *m*/*z*: 291 [M + H]^+^.

##### *N*-(4-(2-Aminocyclopropyl)­phenyl)-4-(cyclopropylethynyl)­benzamide Hydrochloride (**19**)

White solid; yield, 72%. mp >250 °C. Recrystallization solvent: methanol. ^1^H-NMR (DMSO-*d*_6_, 400 MHz): δ_H/ppm_ 0.78 (s, 2H, −C*H*_2_-cyclopropane acetylene), 0.92 (d, 2H, −C*H*_2_-cyclopropane acetylene), 1.16–1.20 (m, 1H, −CH*H*-cyclopropane), 1.35–1.39 (m, 1H, −C*H*H-cyclopropane), 1.55–1.62 (m, 1H, -C*H*-cyclopropane acetylene), 2.29–2.33 (m, 1H, Ar–C*H*-cyclopropane), 2.78–2.80 (m, 1H, -C*H*-NH_3_^+^), 7.14 (d, 2H, benzene ring), 7.50 (d, 2H, benzene ring), 7.70 (d, 2H, benzene ring), 7.91 (d, 2H, benzene ring), 8.38 (bs, 3H, −N*H*_3_^+^), 10.26 (bs, 1H, −CO–N*H*–Ar). MS (ESI), *m*/*z*: 317 [M + H]^+^.

##### *N*-(4-(2-Aminocyclopropyl)­phenyl)-4-(phenylethynyl)­benzamide Hydrochloride (**20**)

White solid; yield, 69%. mp >250 °C. Recrystallization solvent: methanol. ^1^H-NMR (DMSO-*d*_6_, 400 MHz): δ_H_/ppm 1.18–1.21 (m, 1H, −CH*H*-cyclopropane), 1.35–1.40 (m, 1H, −C*H*H-cyclopropane), 2.29–2.33 (m, 1H, Ar–C*H*-cyclopropane), 2.78–2.81 (m, 1H, −C*H*-NH_3_^+^), 7.16 (d, 2H, benzene ring), 7.46–7.47 (m, 3H, benzene ring), 7.60–7.62 (m, 2H, benzene ring), 7.71–7.74 (m, 4H, benzene ring), 8.02 (d, 2H, benzene ring), 8.38 (bs, 3H, −N*H*_3_^+^), 10.34 (bs, 1H, −CO–N*H*–Ar). MS (ESI), *m*/*z*: 353 [M + H]^+^.

##### *N*-(3-(2-Aminocyclopropyl)­phenyl)-4-(prop-1-yn-1-yl)­benzamide Hydrochloride (**21**)

White solid; yield, 75%. mp >250 °C. Recrystallization solvent: methanol. ^1^H-NMR (DMSO-*d*_6_, 400 MHz): δ_H_/ppm 1.14–1.19 (m, 1H, −CH*H*-cyclopropane), 1.26–1.31 (m, H, -C*HH*-cyclopropane), 2.09 (s, 3H, −C*H*_3_), 2.43–2.47 (m, 1H, Ar–C*H*-cyclopropane), 2.79–2.81 (m, 1H, −C*H*–NH_3_^+^), 7.07 (d, 1H, benzene ring), 7.21–7.29 (m, 2H, benzene ring), 7.33–7.35 (m, 1H, benzene ring), 7.53 (d, 2H, benzene ring), 8.03 (d, 2H, benzene ring), 8.39 (bs, 3H, −N*H*_3_^+^), 10.21 (bs, 1H, −CO–N*H*–Ar). MS (ESI), *m*/*z*: 291 [M + H]^+^.

##### *N*-(3-(2-Aminocyclopropyl)­phenyl)-4-(cyclopropylethynyl)­benzamide Hydrochloride (**22**)

White solid; yield, 60%. mp >250 °C. Recrystallization solvent: methanol. ^1^H-NMR (DMSO-*d*_6_, 400 MHz): δ_H/ppm_ 0.79–80 (m, 2H, −C*H*_2_-cyclopropane acetylene), 0.94–0,96 (m, 2H, −C*H*_2_-cyclopropane acetylene), 1.15–1.19 (m, 1H, −CH*H*-cyclopropane), 1.28–1.31 (m, 1H, -C*HH*-cyclopropane), 1.58–1.63 (m, 1H, −C*H*- cyclopropane acetylene), 2.44–2.47 (m, 1H, Ar–C*H*-cyclopropane), 2.81–2.82 (m, 1H, -C*H*-NH_3_^+^), 7.08 (d, 1H, benzene ring), 7.22–7.30 (m, 2H, benzene ring), 7.34 (d, 1H, benzene ring), 7.52–7.53 (d, 2H, benzene ring), 8.03 (d, 2H, benzene ring), 8.38 (bs, 3H, N*H*_3_+), 10.21 (bs, 1H, −CO–N*H*-Ar). MS (ESI), *m*/*z*: 317 [M + H]^+^.

#### General Procedure for the Preparation of the 4-(Alkynyl)­benzoyl Chlorides **27–29**

##### Example: 4-(Prop-1-yn-1-yl)­benzoyl Chloride (**27**)

A mixture of 4-(prop-1-yn-1-yl)­benzoic acid **45** (3 mmol, 480.5 mg, 1 equiv) with an excess of thionyl chloride (SOCl_2_) and two drops of DMF was refluxed at 85 °C for 3 h with nitrogen purge. SOCl_2_ was distilled off, and the crude mixture was purified by flash chromatography with DCM to afford the 4-(prop-1-yn-1-yl)­benzoyl chloride **27** as a low-melting yellowish solid (84%). The acyl chlorides **27–29** were used directly in the subsequent step without prior characterization.

#### General Procedure for the Synthesis of *tert*-Butyl (2-(3- or 4-(3- or 4-Substituted benzamido)­phenyl)­cyclopropyl) Carbamates **30**–**35**, **39**, **40**

##### Example: *tert*-Butyl (2-(4-(4-Propylbenzamido)­phenyl)­cyclopropyl)­carbamate (**31**)

TEA (0.846 mmol, 0.120 mL, 1.5 equiv) and the 4-propylbenzoyl chloride **25** (0.677 mmol, 0.130 mL, 1.2 equiv) were added dropwise to a cooled (0 °C) solution of *tert*-butyl 2-(4-aminophenyl)­cyclopropylcarbamate **23** (0.564 mmol, 140 mg, 1.0 equiv) in dry dichloromethane (5 mL). The mixture was stirred at rt for 2 h; afterward, the reaction was quenched with saturated NaHCO_3_ solution (30 mL) and extracted with AcOEt (3 × 30 mL). The organic phase was washed with saturated NaHCO_3_ and Na_2_CO_3_ solutions and dried over anhydrous Na_2_SO_4_. The solvent was removed under vacuum, and the residue was purified by flash chromatography on silica gel (eluting with AcOEt/*n*-hexane) to provide the pure *tert*-butyl (2-(4-(4- propylbenzamido)­phenyl)­cyclopropyl)­carbamate **31** as white solid (88%). mp 166 °C. Recrystallization solvent: acetonitrile. ^1^H-NMR (CDCl_3_, 400 MHz): δ_H_/ppm 0.97 (t, 3H, –CH_2_CH_2_C*H*_3_), 1.12–1.19 (m, 2H, −C*H*_2_-cyclopropane), 1.48 (s, 9H, –COO­(C*H*_3_)_3_), 1.65–1.72 (m, 2H, −CH_2_C*H*_2_CH_3_), 2.06–2.09 (m, 1H, Ar–C*H*-cyclopropane), 2.68 (t, 2H, −C*H*_2_CH_2_C*H*_3_), 2.72 (m, 1H, -C*H*-NH–COO­(CH_3_)_3_), 4.85 (bs, 1H, -N*H*-COO­(CH_3_)_3_), 7.16 (d, 2H, benzene ring), 7.31 (d, 2H, benzene ring), 7.58 (d, 2H, benzene ring), 7.74 (bs, 1H, Ar–CO–N*H*-Ar), 7.84 (d, 2H, benzene ring). MS (EI) *m*/*z*: 395 [M + H]^+^.

##### *tert*-Butyl (2-(4-(4-Methylbenzamido)­phenyl)­cyclopropyl)­carbamate (**30**)

White solid; yield, 80%. mp 179–181 °C. Recrystallization solvent: acetonitrile/methanol. ^1^H-NMR (CDCl_3_, 400 MHz): δ_H_/ppm 1.14–1.19 (m, 2H, −C*H*_2_- cyclopropane), 1.48 (s, 9H, –COO­(C*H*_3_)_3_), 2.05–2.06 (m, 1H, Ar–C*H*- cyclopropane), 2.45 (s, 3H, −C*H*_3_), 2.69–2.72 (m, 1H, -C*H*-NH–COO­(CH_3_)_3_), 4.85 (bs,1H, -N*H*-COO­(CH_3_)_3_), 7.17 (d, 2H, benzene ring), 7.33 (d, 2H, benzene ring), 7.55 (d, 2H, benzene ring), 7.72 (s, 1H, −CO–N*H*-Ar), 7.82 (d, 2H, benzene ring). MS (ESI), *m*/*z*: 367 [M + H]^+^.

##### *tert*-Butyl (2-(4-(4-Cyanobenzamido)­phenyl)­cyclopropyl)­carbamate (**32**)

White solid; yield, 81%. mp 153–154 °C. Recrystallization solvent: acetonitrile. ^1^H-NMR (CDCl_3_, 400 MHz): δ_H_/ppm 1.17–1.18 (m, 2H, −C*H*_2_- cyclopropane), 1.48 (s, 9H, –COO­(C*H*_3_)_3_), 2.07–2.11 (m, 1H, Ar–C*H*-cyclopropane), 2.71–2.74 (m, 1H, -C*H*-NH–COO­(CH_3_)_3_), 4.86 (bs,1H, –N*H*-COO­(CH_3_)_3_), 7.19 (d, 2H, benzene ring), 7.54 (d, 2H, benzene ring), 7.73 (bs, 1H, −CO–N*H*-Ar) 7.82 (d, 2H, benzene ring), 8.00 (d, 2H, benzene ring). MS (ESI), *m*/*z*: 378 [M + H]^+^.

##### *tert*-Butyl (2-(4-(4-(Prop-1-yn-1-yl)­benzamido)­phenyl)­cyclopropyl)­carbamate (**33**)

White solid; yield, 74%. mp 142–143 °C. Recrystallization solvent: acetonitrile. ^1^H-NMR (CDCl_3_, 400 MHz): δ_H_/ppm 1.16–1.19 (m, 2H, −C*H*_2_-cyclopropane), 1.47 (s, 9H, –COO­(C*H*_3_)_3_), 2.07 (s, 3H, −C*H*_3_), 2.12–2.14 (m, 1H, -C*H*-NH–COO­(CH_3_)_3_), 2.69–2.71 (m, 1H, Ar–C*H*-cyclopropane), 4.91 (bs, 1H, -N*H*-COO­(CH_3_)_3_), 7.12 (d, 2H, benzene ring), 7.48 (d, 2H, benzene ring), 7.66 (d, 2H, benzene ring), 7.72 (s, 1H, −CO–N*H*-Ar), 7.91 (d, 2H, benzene ring). MS (ESI), *m*/*z*: 391 [M + H]^+^.

##### *tert*-Butyl (2-(4-(4-(Cyclopropylethynyl)­benzamido)­phenyl)­cyclopropyl)­carbamate (**34**)

White solid; yield, 75%. mp 169–170 °C. Recrystallization solvent: acetonitrile. ^1^H-NMR (CDCl_3_, 400 MHz): δ_H_/ppm 0.77 (d, 2H, −C*H*_2_-cyclopropane acetylene), 0.91 (d, 2H, −C*H*_2_-cyclopropane acetylene), 1.15–1.18 (m, 2H, −C*H*_2_-cyclopropane), 1.47 (s, 9H, –COO­(C*H*_3_)_3_), 1.55–1.60 (m, 1H, -C*H*- cyclopropane acetylene), 2.08–2.11 (m, 1H, Ar–C*H*- cyclopropane), 2.70–2.73 (m, 1H, -C*H*-NH–COO­(CH_3_)_3_), 4.87 (bs, 1H, -N*H*-COO­(CH_3_)_3_), 7.17 (d, 2H, benzene ring), 7.49 (d, 2H, benzene ring), 7.61 (d, 2H, benzene ring), 7.72 (s, 1H, −CO–N*H*-Ar) 7.89 (d, 2H, benzene ring). MS (ESI), *m*/*z*: 417 [M + H]^+^.

##### *tert*-Butyl (2-(4-(4-(Phenylethynyl)­benzamido)­phenyl)­cyclopropyl)­carbamate (**35**)

White solid; yield, 70%. mp 195–196 °C. Recrystallization solvent: acetonitrile/methanol. ^1^H-NMR (CDCl_3_, 400 MHz): δ_H_/ppm 1.16–1.20 (m, 2H, −C*H*_2_- cyclopropane), 1.48 (s, 9H, –COO­(C*H*_3_)_3_), 2.05–2.27 (m, 1H, Ar–C*H*-cyclopropane), 2.71–2.74 (m, 1H, -C*H*-NH–COO­(CH_3_)_3_), 4.85 (bs, 1H, –N*H*–COO­(CH_3_)_3_), 7.18 (d, 2H, benzene ring), 7.39–7–41 (m, 3H, benzene ring), 7.55–7.59 (m, 4H, benzene ring), 7.66 (d, 2H, benzene ring), 7.76 (bs, 1H, −CO–N*H*-Ar), 7.87 (d, 2H, benzene ring). MS (ESI), *m*/*z*: 453 [M + H]^+^.

##### *tert*-Butyl (2-(3-(4-(Prop-1-yn-1-yl)­benzamido)­phenyl)­cyclopropyl)­carbamate (**39**)

White solid; yield, 69%. mp 149–150 °C. Recrystallization solvent: acetonitrile. ^1^H-NMR (CDCl_3_, 400 MHz): δ_H_/ppm 1.12 (d, 2H, −C*H*_2_-cyclopropane), 1.47 (s, 9H, –COO­(C*H*_3_)_3_), 2.01 (s, 3H, −C*H*_3_), 2.17–2.22 (m, 1H, Ar–C*H*-cyclopropane), 2.70–2.73 (m, 1H, -C*H*-NH–COO­(CH_3_)_3_), 4.89 (bs, 1H, -N*H*-COO­(CH_3_)_3_), 7.14 (d, 1H, benzene ring), 7.19–7.22 (m, 2H, benzene ring), 7.39–7.42 (m, 1H, benzene ring), 7.60 (d, 2H, benzene ring), 7.74 (s, 1H, −CO–N*H*-Ar), 7.99 (d, 2H, benzene ring). MS (ESI), *m*/*z*: 391 [M + H]^+^.

##### *tert*-Butyl (2-(3-(4-(Cyclopropylethynyl)­benzamido)­phenyl)­cyclopropyl)­carbamate (**40**)

White solid; yield, 73%. mp 161–162 °C. Recrystallization solvent: acetonitrile. ^1^H-NMR (CDCl_3_, 400 MHz): δ_H_/ppm 0.80 (d, 2H, −C*H*_2_-cyclopropane acetylene), 0.92 (d, 2H, −C*H*_2_-cyclopropane acetylene), 1.14–1.19 (m, 2H, −C*H*_2_-cyclopropane), 1.48 (s, 9H, –COO­(C*H*_3_)_3_), 1.55–1.58 (m, 1H, -C*H*-cyclopropane acetylene), 2.09–2.12 (m, 1H, Ar–C*H*-cyclopropane), 2.72–2.76 (m, 1H, −C*H*–NH–COO­(CH_3_)_3_), 4.89 (bs, 1H, −N*H*–COO­(CH_3_)_3_), 7.15 (d, 1H, benzene ring), 7.29–7.31 (m, 2H, benzene ring), 7.45 (d, 1H, benzene ring), 7.62 (d, 2H, benzene ring), 7.79 (s, 1H, −CO–N*H*-Ar), 7.99 (d, 2H, benzene ring). MS (ESI), *m*/*z*: 417 [M + H]^+^.

##### *tert*-Butyl (2-(4-(3-ethynylbenzamido)­phenyl)­cyclopropyl)­carbamate (**37**)

3-Ethynylbenzoic acid **36** (0.93 mmol, 135.4 mg, 1.15 equiv), 1-ethyl-3-(3-(dimethylamino)­propyl)­carbodiimide hydrochloride (EDCI) (1.13 mmol, 216.2 mg, 1.4 equiv), hydroxybenzotriazole (HOBt) (152.4 mg, 1.13 mmol, 1.4 equiv) and TEA (0.43 mL, 3.06 mmol, 3.8 equiv) were sequentially added to a solution of **23** (200 mg, 0.805 mmol, 1.0 equiv) in dry DMF (4.5 mL). The resulting mixture was stirred for approximately 7 h at rt and, after completion of the reaction, quenched with NaHCO_3_ saturated solution (40 mL). The aqueous solution was extracted with AcOEt (4 × 25 mL); washed with 0.1 N KHSO_4_ solution (2 × 10 mL), NaHCO_3_ saturated solution (3 × 10 mL), and brine (3 × 5 mL); dried over anhydrous Na_2_SO_4_ and finally concentrated under vacuum. The crude product was purified by column chromatography on silica gel eluting with AcOEt/*n*-hexane 25:75 (*v/v*) mixture to afford **37** as a white solid (193 mg, 64%). mp 111–112 °C. Recrystallization solvent: toluene. ^1^H-NMR (CDCl_3_, 400 MHz): δ_H_/ppm 1.07–1.10 (m, 2H, −C*H*_2_-cyclopropane), 1.39 (s, 9H, –COO*(CH*_3_)_3_), 1.94–1.98 (m, 1H, Ar–C*H*-cyclopropane), 2.62–2.66 (m, 1H, −C*H*–NH–COO­(CH_3_)_3_), 3.08 (s, 1H, C*H* acetylene), 4.77 (s, 1H, -N*H*-COO­(CH_3_)_3_), 7.08 (d, 2H, benzene ring), 7.39 (t, 1H, benzene ring), 7.45 (d, 2H, benzene ring), 7.59 (d, 1H, benzene ring), 7.64 (s, 1H, Ar–CO–N*H*-Ar), 7.79 (d, 1H, benzene ring), 7.89 (s, 1H, benzene ring). MS (ESI), *m*/*z*: 377 [M + H]^+^.

#### General Procedure for the Synthesis of the Methyl 4-(Alkynyl)­benzoates **42**–**44**

##### Example: Methyl 4-Cyclopropylethynylbenzoate (**43**). Methyl 4-Iodobenzoate (**41**)

(2.50 mmol, 655 mg, 1 equiv), TEA (25 mmol, 3.5 mL, 10 equiv), CuI (5 mol %), Bis­(triphenylphosphine)­palladium­(II) dichloride (PdCl_2_(PPh_3_)_2_) (5 mol %), and ethynylcyclopropane (3.25 mmol, 214.8 mg, 1.3 equiv) were dissolved in dry DMF (10 mL). The mixture was stirred at 70 °C overnight. Upon completion, the reaction was quenched with AcOEt, filtered over Celite, and the organic phases were combined, washed with water and brine, and dried over MgSO_4_. After solvent evaporation, the residue was purified via flash chromatography (AcOEt/*n*-hexane) to give methyl 4-cyclopropylethynylbenzoate (**43**) as a white solid (86%). mp 46–47 °C. Recrystallization solvent: *n*-hexane. ^1^H-NMR (CDCl_3_, 400 MHz): δ_H_/ppm 0.75–0.77 (m, 2H, −C*H*_2_-cyclopropane), 0.82–0.85 (m, 2H, −C*H*_2_-cyclopropane), 1.38–1.42 (m, 1H, C*H* cyclopropane), 3.83 (s, 3H, −COOC*H*_3_), 7.34 (d, 2H, benzene ring), 7.88 (d, 2H, benzene ring). MS (ESI), *m*/*z*: 201 [M + H]^+^.

##### Methyl 4-(Prop-1-yn-1-yl)­benzoate (**42**)

White solid; yield, 69%. mp 58–59 °C. Recrystallization solvent: *n*-hexane. ^1^H-NMR (CDCl_3_, 400 MHz): δ_H_/ppm 2.08 (s, 3H, −C*H*_3_), 3.91 (s, 3H, −COOC*H*_3_), 7.44 (d, 2H, benzene ring), 7.95 (d, 2H, benzene ring). MS (ESI), *m*/*z*: 175 [M + H]^+^.

##### Methyl 4-(phenylethynyl)­benzoate (**44**)

White solid; yield, 76%. mp 62–65 °C. Recrystallization solvent: *n*-hexane. ^1^H-NMR (CDCl_3_, 400 MHz): δ_H_/ppm 3.93 (s, 3H, −COOC*H*_3_), 7.36–7.37 (m, 3H, benzene ring), 7.55 (m, 2H, benzene ring), 7.59 (d, 2H, benzene ring), 8.02 (d, 2H, benzene ring). MS (ESI), *m*/*z*: 237 [M + H]^+^.

#### General Procedure for the Preparation of the 4-(Alkynyl)­benzoic Acids **45–47**

##### Example: 4-(Phenylethynyl)­benzoic Acid (**47**)

A 2 N LiOH (6.81 mmol, 0.285 mL, 3 equiv) solution was added to a solution of methyl 4-(phenylethynyl)­benzoate **44** (2.27 mmol, 536 mg, 1 equiv) dissolved in 60 mL of methanol/water (3:1), and the mixture was stirred at room temperature (rt) for 5 min, and heated under reflux overnight. The reaction mixture was allowed to cool to rt, and the solvent was evaporated under reduced pressure. The residue was suspended in water, and the suspension was acidified with 1 N HCl solution (pH = 2–4), and stirred at rt for 30 min. The suspension obtained was filtered, washed with water, and dried to obtain the 4-(phenylethynyl)­benzoic acid **33** as a white solid (74%). mp 218–220 °C. Recrystallization solvent: acetonitrile/methanol. ^1^H-NMR (CDCl_3_, 400 MHz): δ_H_/ppm 7.45–7.47 (m, 3H, benzene ring), 7.59–7.61 (m, 2H, benzene ring), 7.67 (d, 2H, benzene ring), 7.97 (d, 2H, benzene ring), 13.19 (broad s, 1H, −COO*H*). MS (ESI), *m*/*z*: 221 [M – H]^−^.

##### 4-(Prop-1-yn-1-yl)­benzoic Acid (**45**)

White solid; yield, 67%. mp 121–123 °C. Recrystallization solvent: toluene. ^1^H-NMR (CDCl_3_, 400 MHz): δ_H_/ppm 1.98 (s, 3H, −C*H*_3_), 7.48 (d, 2H, benzene ring), 7.91 (d, 2H, benzene ring), 12.95 (broad s, 1H, −COO*H*). MS (ESI), *m*/*z*: 159 [M – H]^−^.

##### 4-(Cyclopropylethynyl)­benzoic Acid (**46**)

White solid; yield, 76%. mp 220–222 °C. Recrystallization solvent: acetonitrile/methanol. ^1^H-NMR (CDCl_3_, 400 MHz): δ_H/ppm_ 0.75–0.77 (m, 2H, −C*H*_2_-cyclopropane), 0.90–0.92 (m, 2H, −C*H*_2_-cyclopropane), 1.55–1.58 (m, 1H, C*H* cyclopropane), 7.44 (d, 2H, benzene ring), 7.87 (d, 2H, benzene ring), 13.04 (broad s, 1H, −COO*H*). MS (ESI), *m*/*z*: 185 [M – H]^−^.

### LSD1-CoREST Binding and Inhibition Assays

Protein expression, thermal stability, and inhibition of human LSD1-CoREST were performed using established protocols.^37^ IC_50_ determinations were performed in a buffer containing 0.2 μM LSD1-CoREST, 50 mM HEPES pH 7.5, 0.125 mM Amplex Red, and 0.015 mg/mL peroxidase and 10 μM H3dimethylK4 21 aa peptide. LSD1-CoREST (0.4 μM) was preincubated with the inhibitor at 25 °C for 10 min in the presence of 5% DMSO. The reaction was initiated by adding an equal volume of the substrate solution to the enzyme–inhibitor mixture, and resorufin fluorescence (excitation at 530–570 nm and emission at 580–590 nm) was detected with a Clariostar plate reader.

### Newly Transformed Schistosomula (NTS), Juvenile and Adult Worms

All experiments were performed at the Swiss Tropical and Public Health Institute (Swiss TPH) in Allschwil. Approval was given by the veterinary authorities of the Canton Basel-Landschaft (permission no. 545) based on Swiss cantonal and national regulations. The life cycle is maintained at the Swiss TPH as described previously.^44^ Cercariae were obtained from infected snails by exposing them to a strong light source for 3–4 h in pond water. Shed cercariae were mechanically transformed into NTS, incubated at 37 °C with 5% CO_2_ in medium 199, supplemented with 5% FCS and 1% penicillin/streptomycin, for at least 12 h to a maximum of 24 h before use. To obtain juvenile worms, NTS were transferred to 10 mL Human Serum, collected from Blutspendezentrum Basel (Switzerland) and 40 mL Panserin 401 serum free medium (Pan biotech, Germany) and 500 μL penicillin/streptomycin. Adult worms were collected by dissecting the mesenteric veins of infected mice at day 49 postinfection, then incubated in supplemented RPMI medium (5% FCS, 100 U/mL penicillin, and 100 μg/mL streptomycin) at 37 °C with 5% CO_2_ until needed.

### In Vitro Phenotypic Screening Assays

For NTS and adult worms, transparent flat-bottom 96- and 24-well plates were used, respectively (Sarstedt, Switzerland). For juvenile worms, 48-well plates were used with 3–6 worms per plate and RPMI medium with 5% FCS and 1% penicillin/streptomycin. Compounds were initially tested at 20 and 10 μM in triplicate on NTS and repeated once; each well contained 30–40 NTS. Phenotypic reference points such as motility, morphology, and granularity were used to score incubated parasites’ overall viability (scores from 0 to 3).^44^ Parasites were observed via microscopic readout 72 h postincubation; compounds showing >50% activity at 10–20 μM were further tested at lower concentrations for IC_50_ determination (Calcusyn software version 2.0). For speed of action experiments, NTS and juvenile worms were evaluated at 4, 24, 48, and 72 h. Identified hits from the NTS screening were tested on adult worms. At least three worms (both sexes) were incubated with RPMI 1640 supplemented with 5% (v/v) FCS and 1% (v/v) penicillin/streptomycin at 37 °C with 5% CO_2_ for 72 h at concentrations of 20 and 10 μM.^44^ The experiment was conducted in duplicate and repeated; standard deviations were calculated from two wells. Worms were monitored for pairing, and at 4, 8, 24, 48, and 72 h for egg laying, and eggs in the wells were counted. For all in vitro assays, negative controls (DMSO at the highest tested concentration) were included.

### Determination of Cytotoxicity on Human Non-transformed Cells

Human commercial lung diploid fibroblasts (MRC-5), and diploid retinal epithelium cells (RPE) were obtained from the American Type Culture Collection (ATCC) and kindly provided by Dr Francesca Degrassi (CNR IBPM) and maintained in minimal essential medium or DMEM-F12 medium supplemented with fetal calf serum, antibiotics, and nonessential amino acids. Cells were grown at 37 °C, in a humidified atmosphere, with 5% CO_2_. For experiments, cells were seeded in 200 μL of complete in each well of a 96-well microtiter plate. After 24 h, compounds were added at 5, 10, 25, and 50 μM for 72 h in sextuplicate. MTT was added (0.5 mg/mL), incubated for 4 h at 37 °C, and dissolved in 200 μL of isopropyl alcohol. Absorbance was measured at 570 nm (ELISA reader, DASIT), and cell viability was calculated as

$$viability=\left(\frac{OD\;of\;treated\;cells}{OD\;of\;control\;cells}\right)\times 100$$

The IC_50_ (concentration of compounds causing 50% inhibition of cell viability) was calculated for each compound.

### Statistical Analysis

Given the exploratory objective of this initial screen, aimed at identifying compounds with promising activity for follow-up studies, the analysis was primarily descriptive in nature, and formal inferential statistical testing was not conducted.

## Supplementary Material



## Notes

(a) IC_50_ values of TCP-based human LSD1 inhibitors **1**–**13**; (b) correlation between human LSD1 inhibition (IC_50_ values) and activity in NTS (IC_50_ values) by **2**, **5**, **15**, **18**, and **19**; (c) speed of action assays in NTS; (d) speed of action assays in juvenile forms; (e) elemental analyzes for compounds **14**–**22** (PDF)

## △

School of Pharmacy and Biomolecular Sciences, Liverpool John Moores University, Liverpool, L3 3AF, United Kingdom

## ○

E.F. and G.S.H., equal contribution. A.Mai, D.R. and R.F. contributed to conceptualization, supervision, writing—original draft, review and editing; E.F., G.S.H., C.Z., C.L., G.F., A.I., J.C., and C.H. contributed to experiments, investigation, data analysis, visualization; D.R., A.G., S.V., D.T., A.Mattevi, and J.K. contributed to ideas, assisted in data analysis, and participated in manuscript drafting and proofreading. All authors approved the submitted manuscript.
