# Supplementary material for: Tranylcypromine-Based LSD1 Inhibitors as Useful Agents to Reduce Viability of
Source: ACS Infect Dis. 2025 Jul 2;11(8):2178–89. doi: 10.1021/acsinfecdis.5c00224 (PMC12340956; doi:10.1021/acsinfecdis.5c00224)
Supplement: Supplementary file 1 [file id5c00224_si_001.pdf]

# Tranylcypromine-based LSD1 Inhibitors as Useful Agents to Reduce Viability of *Schistosoma mansoni*

Emanuele Fabbrizi,[^,‖] Gebremedhin Solomon Hailu,[ϕ,‖] A. Ganesan,[¥] Rossella Fioravanti,[^,*] Clemens Zwergel,[^] Chiara Lambona,[^] Sergio Valente,[^] Giulia Fianco,[†] Angela Iuzzolino,[†] Daniela Trisciuoglio,[†] Jonatan Caroli,[#] Andrea Mattevi,[#] Cécile Häberli,[+] Jennifer Keiser,[+] Dante Rotili,[°,§,*] and Antonello Mai[^]

[^]*Department of Drug Chemistry and Technologies, Sapienza University of Rome, Piazzale Aldo Moro 5, 00185 Rome, Italy*
[ϕ]*School of Pharmacy, Mekelle University, Mekelle, Ethiopia*
[¥]*School of Pharmacy, University of East Anglia, Norwich NR4 7TJ, United Kingdom*
[†]*Institute of Molecular Biology and Pathology, National Research Council (CNR), 00185 Rome, Italy*
[#]*Department of Biology and Biotechnology, University of Pavia, Via Ferrata 9, 27100 Pavia, Italy*
[+]*Swiss Tropical and Public Health Institute, 4002 Allschwil, Switzerland; University of Basel, 4001 Basel, Switzerland*
°*Department of Science, Roma Tre University of Rome, Viale Guglielmo Marconi 446, 00146 Rome, Italy*
[§]*Biostructures and Biosystems National Institute (INBB), Via dei Carpegna 19, 00165 Rome, Italy*

[‖]E.F. and G.S.H., equal contribution.

*Corresponding authors: R.F.: rossella.fioravanti@uniroma1.it; D.R.: dante.rotili@uniroma3.it

## Supporting Information

**Content:**

**Table S1.** $IC_{50}$ values of TCP-based human LSD1 inhibitors **1-13**. p. S2

**Figure S1.** Correlation between human LSD1 inhibition ($IC_{50}$ values) and activity in NTS ($IC_{50}$ values) by **2**, **5**, **15**, **18**, and **19**. p. S3

**Table S2.** Speed of action assays in NTS. p. S4

**Table S3.** Speed of action assays in *S. mansoni* juvenile forms. p. S5

**Table S4.** Elemental analyses for compounds **14-22**. p. S6

**Table S1.** $IC_{50}$ values of TCP-based human LSD1 inhibitors **1-13**

| compd | $IC_{50}$, µM | Ref. |
|---|---|---|
| **1** | <1 | 1 |
| **2** | 0.05 ± 0.01 | 2 |
| **3** | 2.00 ± 0.53 | 3 |
| **4** | 0.34 ± 0.11 | 3 |
| **5** | 0.09 ± 0.01 | 2 |
| **6** | 0.05 ± 0.004 | 4 |
| **7** | 0.09 ± 0.01 | 4 |
| **8** | 0.04 ± 0.01 | 2 |
| **9** | 0.30 ± 0.03 | 4 |
| **10** | 0.06 ± 0.01 | 4 |
| **11** | 0.15 ± 0.03 | 2 |
| **12** | 0.19 ± 0.03 | 2 |
| **13** | 0.08 ± 0.01 | 2 |

## References

(1) Rotili, D.; Tomassi, S.; Conte, M.; Benedetti, R.; Tortorici, M.; Ciossani, G.; Valente, S.; Marrocco, B.; Labella, D.; Novellino, E.; Mattevi, A.; Altucci, L.; Tumber, A.; Yapp, C.; King, O. N.; Hopkinson, R. J.; Kawamura, A.; Schofield, C. J.; Mai, A. Pan-Histone Demethylase Inhibitors Simultaneously Targeting Jumonji C and Lysine-Specific Demethylases Display High Anticancer Activities. *J Med Chem* **2014,** 57, 42-55.

(2) Fioravanti, R.; Romanelli, A.; Mautone, N.; Di Bello, E.; Rovere, A.; Corinti, D.; Zwergel, C.; Valente, S.; Rotili, D.; Botrugno, O. A.; Dessanti, P.; Vultaggio, S.; Vianello, P.; Cappa, A.; Binda, C.; Mattevi, A.; Minucci, S.; Mercurio, C.; Varasi, M.; Mai, A. Tranylcypromine-Based Lsd1 Inhibitors: Structure-Activity Relationships, Antiproliferative Effects in Leukemia, and Gene Target Modulation. *ChemMedChem* **2020,** 15, 643-658.

(3) Fioravanti, R.; Rodriguez, V.; Caroli, J.; Chianese, U.; Benedetti, R.; Di Bello, E.; Noce, B.; Zwergel, C.; Corinti, D.; Vina, D.; Altucci, L.; Mattevi, A.; Valente, S.; Mai, A. Heterocycle-Containing Tranylcypromine Derivatives Endowed with High Anti-Lsd1 Activity. *J Enzyme Inhib Med Chem* **2022,** 37, 973-985.

(4) Rodriguez, V.; Valente, S.; Rovida, S.; Rotili, D.; Stazi, G.; Lucidi, A.; Ciossani, G.; Mattevi, A.; Botrugno, O. A.; Dessanti, P.; Mercurio, C.; Vianello, P.; Minucci, S.; Varasi, M.; Mai, A. Pyrrole- and Indole-Containing Tranylcypromine Derivatives as Novel Lysine-Specific Demethylase 1 Inhibitors Active on Cancer Cells. *MedChemComm* **2015,** 6, 665-670.

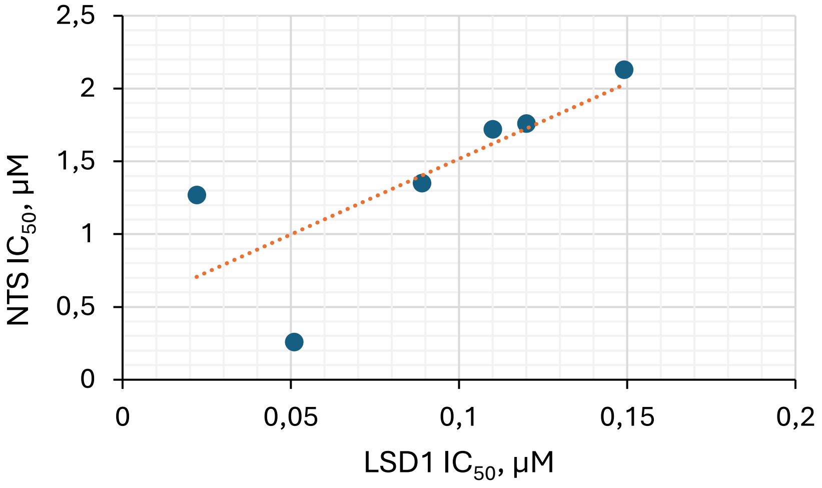


**Figure S1.** Correlation between human LSD1 inhibition ($IC_{50}$ values) and activity in NTS (IC50 values) by **2**, **5**, **15**, **18**, and **19**.

**Table S2.** Speed of action assays in NTS.

| compd | Percentage of effect (SD) | | | | | | | |
|---|---|---|---|---|---|---|---|---|
| | 4 h | | 24 h | | 48 h | | 72 h | |
| | 20 µM | 10 µM | 20 µM | 10 µM | 20 µM | 10 µM | 20 µM | 10 µM |
| **2** | 38.46 (3.8) | 30.77 (0) | 82.14 (3.6) | 78.57 (0) | 93.88 (2) | 87.76 (4.1) | 100 (0) | 89.80 (6.1) |
| **15** | 38.46 (0) | 21.15 (1.9) | 57.14 (3.6) | 50 (0) | 91.84 (0) | 51.02 (0) | 100 (0) | 71.43 (0) |
| **18** | 30.77 (0) | 26.92 (0) | 75 (3.6) | 51.79 (1.8) | 87.76 (0) | 81.63 (2) | 100 (0) | 85.81 (2) |
| **19** | 38.46 (3.8) | 30.77 (3.8) | 78.57 (0) | 48.21 (1.8) | 100 (0) | 87.76 (0) | 100 (0) | 93.88 (2) |
| MC3935 | 23.08 (0) | 21.15 (1.9) | 41.07 (1.8) | 41.07 (1.8) | 57.14 (6.1) | 34.69 (4.1) | 59.18 (4.1) | 51.02 (0) |

**Table S3.** Speed of action assays in *S. mansoni* juvenile forms.

| **compd** | **Percentage of effect (SD)** | | | | | | | | | | | |
|---|---|---|---|---|---|---|---|---|---|---|---|---|
| | **4 h** | | | **24 h** | | | **48 h** | | | **72 h** | | |
| | **20 µM** | **10 µM** | **1 µM** | **20 µM** | **10 µM** | **1 µM** | **20 µM** | **10 µM** | **1 µM** | **20 µM** | **10 µM** | **1 µM** |
| **2** | 43.64 (1.8) | 23.33 (3.3) | 20 (0) | 57.69 (3.8) | 37.93 (0) | 24.14 (6.9) | 56.25 (2.1) | 36.45 (1.9) | 32.69 (1.9) | 70.83 (4.2) | 39.11 (2.1) | 39 (1.7) |
| **15** | 45.45 (0) | 33.33 (0) | 23.33 (3.3) | 48.08 (1.9) | 34.48 (3.4) | 24.14 (0) | 50 (0) | 42.31 (3.8) | 34.62 (3.8) | 70.83 (0) | 44.55 (7.3) | 40.96 (0.2) |
| **18** | 43.64 (1.8) | 20 (0) | 20 (0) | 46.15 (0) | 31.03 (0) | 24.14 (6.9) | 47.92 (2.1) | 42.31 (0) | 34.62 (0) | 70.83 (0) | 44.77 (0.3) | 42.81 (1.6) |
| **19** | 41.82 (3.6) | 20 (0) | 20 (0) | 40.38 (1.9) | 34.48 (3.4) | 27.59 (3.4) | 47.92 (6.3) | 46.15 (3.8) | 36.54 (1.9) | 81.25 (6.3) | 46.73 (2.3) | 43.03 (6) |
| MC3935 | 41.82 (3.6) | 23.33 (3.3) | 16.67 (3.3) | 38.46 (3.8) | 31.03 (0) | 17.24 (0) | 39.58 (2.1) | 32.69 (1.9) | 26.92 (0) | 56.25 (2.1) | 39 (1.7) | 31.48 (1.9) |

**Table S4.** Elemental analyses for compounds **14-22**.

| Cmpd | Formula | MW | Calculated, % | | | Found, % | | |
|---|---|---|---|---|---|---|---|---|
| | | | C | H | N | C | H | N |
| **14** | $C_{17}H_{18}N_2O$ | 266.34 | 76.66 | 6.81 | 10.52 | 76.22 | 6.74 | 10.88 |
| **15** | $C_{19}H_{22}N_2O$ | 294.40 | 77.52 | 7.53 | 9.52 | 77.79 | 7.61 | 9.19 |
| **16** | $C_{18}H_{16}N_2O$ | 276.34 | 78.24 | 5.84 | 10.14 | 77.95 | 5.72 | 10.37 |
| **17** | $C_{17}H_{15}N_3O$ | 277.33 | 73.63 | 5.45 | 15.15 | 73.45 | 5.44 | 15.39 |
| **18** | $C_{19}H_{18}N_2O$ | 290.37 | 78.59 | 6.25 | 9.65 | 78.92 | 6.44 | 9.36 |
| **19** | $C_{21}H_{20}N_2O$ | 316.40 | 79.72 | 6.37 | 8.85 | 79.48 | 6.29 | 9.11 |
| **20** | $C_{24}H_{20}N_2O$ | 352.44 | 81.79 | 5.72 | 7.95 | 82.10 | 5.88 | 7.62 |
| **21** | $C_{19}H_{18}N_2O$ | 290.37 | 78.59 | 6.25 | 9.65 | 78.26 | 6.14 | 10.02 |
| **22** | $C_{21}H_{20}N_2O$ | 316.40 | 79.72 | 6.37 | 8.85 | 79.51 | 6.44 | 9.14 |
